# Painless Thoracic Herpes Zoster Eruption in an 88-Year-Old Woman With Advanced Chronic Kidney Disease: A Case Report

**DOI:** 10.7759/cureus.115723

**Published:** 2026-09-03

**Authors:** Yasser Bouabdella

**Affiliations:** 1 Cardiology, St. Vincentius-Kliniken, Augsburg, DEU

**Keywords:** chronic kidney disease, herpes zoster, older-aged patients, pain, postherpetic neuralgia, varicella-zoster virus

## Abstract

Herpes zoster (HZ) typically presents with a unilateral vesicular eruption accompanied by neuropathic pain. Although pain is a characteristic feature, painless presentations may occur and can create a diagnostic challenge. We report the case of an 88-year-old woman with advanced chronic kidney disease (CKD) who developed a unilateral vesicular eruption involving the right thoracic dermatome without pain at any stage of the disease. She was not receiving immunosuppressive therapy and had no known diabetes mellitus, previous HZ, or neurological disorder. The diagnosis was made clinically based on the characteristic unilateral dermatomal distribution and morphology of the lesions. Renally adjusted oral valacyclovir was administered for seven days, with complete resolution of the cutaneous lesions within approximately one week. No postherpetic neuralgia (PHN) developed, and the patient remained completely pain-free during follow-up. This case highlights that the absence of pain should not exclude HZ, even in very elderly patients with advanced CKD. Careful recognition of the characteristic dermatomal morphology remains essential for timely diagnosis and treatment.

## Introduction

Herpes zoster (HZ) results from reactivation of latent varicella zoster virus (VZV) in sensory ganglia and typically presents as a unilateral vesicular eruption in a dermatomal distribution. Neuropathic pain is a prominent feature of the disease and may precede, accompany, or persist after the rash. Advanced age increases the risk of HZ and its complications, including postherpetic neuralgia (PHN) [1-3].

Chronic kidney disease (CKD) is associated with immune dysfunction and an increased susceptibility to infections, including HZ. The risk may be further increased by factors such as dialysis or immunosuppressive therapy [4,5].

Complete absence of pain, however, is an unusual presentation of HZ. Painless cases have occasionally been reported, but remain uncommon [6]. Here, we describe a clinically diagnosed, completely painless thoracic HZ eruption in an 88-year-old woman with advanced CKD who was not receiving immunosuppressive therapy.

## Case presentation

An 88-year-old woman developed a unilateral vesicular eruption involving the right lateral and posterior thoracic wall during hospitalization for heart failure. The eruption consisted of grouped vesicles with areas of crusting on an erythematous background and followed a characteristic unilateral dermatomal distribution, clinically estimated to correspond approximately to the right T9 dermatome (Figures 1, 2).

**Figure 1:**
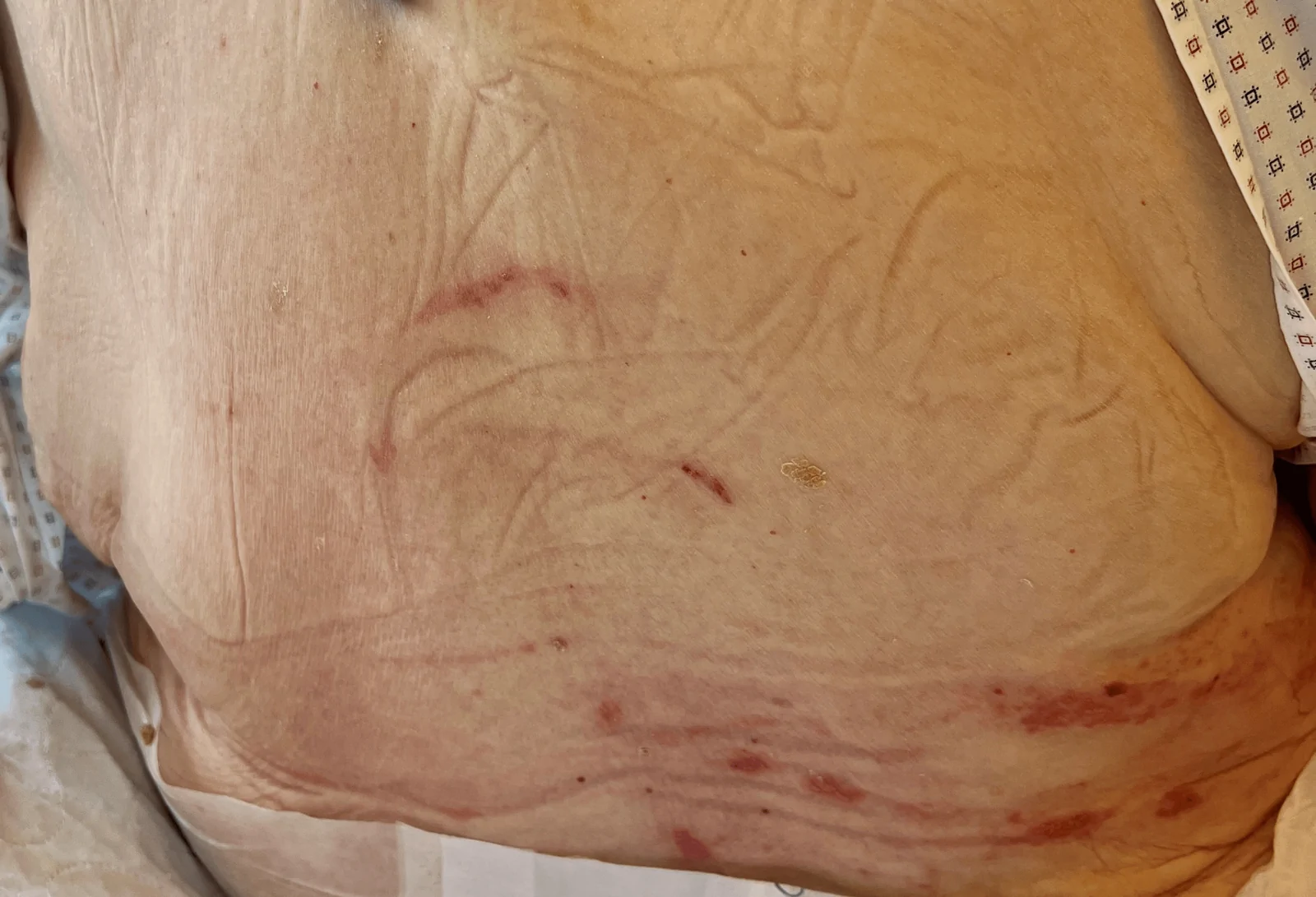
Posterior view of the painless herpes zoster eruption. The photograph demonstrates the unilateral distribution of the lesions over the right posterior thoracic region, clinically consistent with approximately the T9 dermatome.

**Figure 2:**
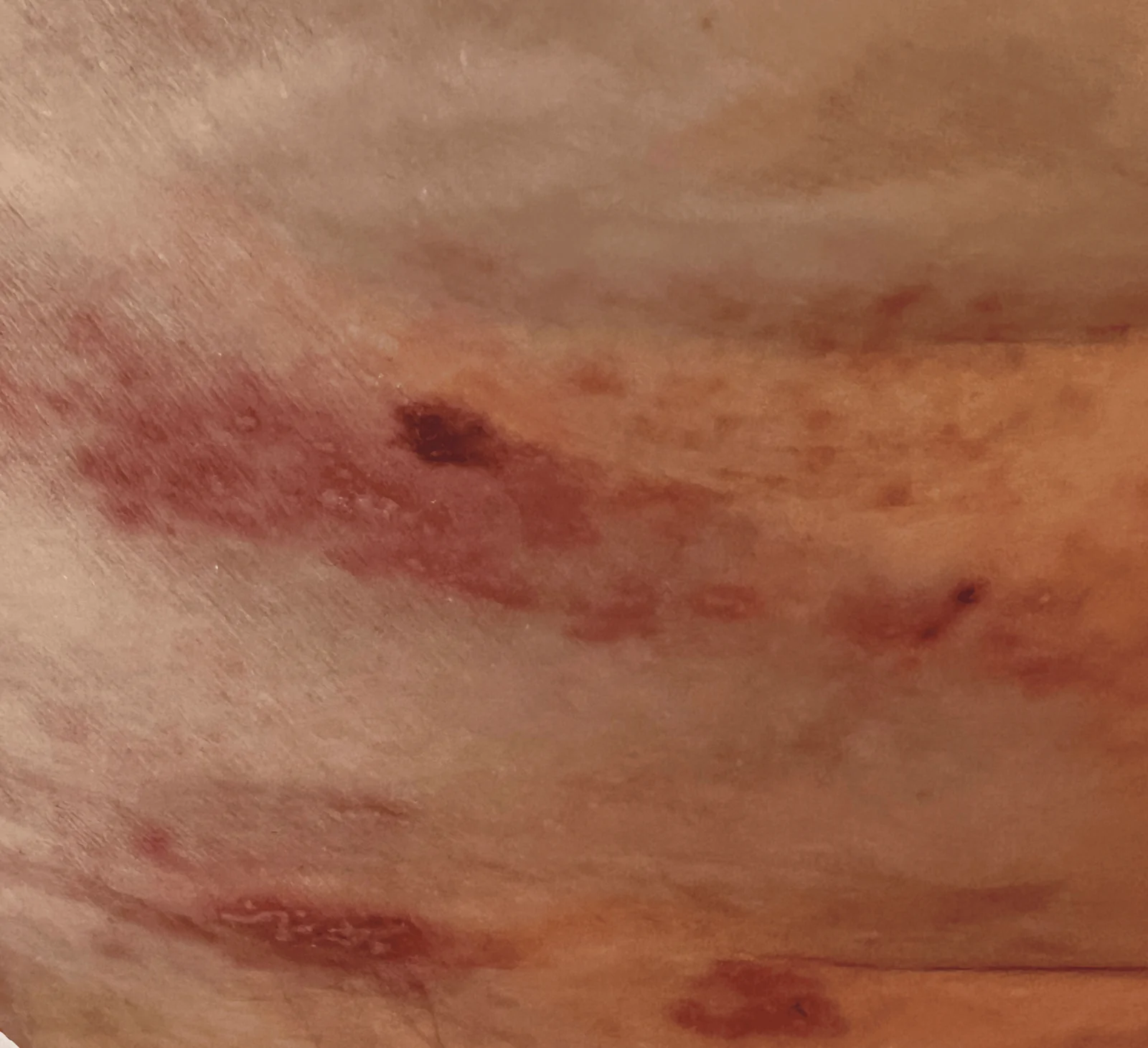
Close-up view of the painless herpes zoster lesions. The photograph shows erythematous lesions with vesicular and crusted components.

Remarkably, the patient reported no pain at any stage. She denied spontaneous pain, burning, pruritus, and dysesthesia. Palpation of the affected area did not elicit pain. Pain intensity was consistently assessed as 0/10 throughout the acute episode and during follow-up.

Her medical history was notable for advanced CKD. Serum creatinine was 2.7 mg/dL at admission and decreased to 2.5 mg/dL during hospitalization. Her estimated glomerular filtration rate was approximately 11 mL/min/1.73 m² initially and subsequently improved to approximately 18 mL/min/1.73 m².

The patient was not receiving immunosuppressive or immunomodulatory therapy. There was no known history of diabetes mellitus, previous HZ, or neurological disease. Her HZ vaccination status was unknown.

The diagnosis of HZ was established clinically based on the characteristic morphology and unilateral dermatomal distribution of the cutaneous eruption. Although the absence of pain represented an unusual clinical feature, the morphology and distribution of the rash were considered sufficiently characteristic during routine care that additional laboratory confirmation was not performed. Consequently, VZV PCR was not available for this case. No alternative confirmatory testing, including direct fluorescent antibody testing or serological testing, was performed.

The patient was treated with renally adjusted oral valacyclovir at a dose of 500 mg once daily for seven days. Treatment was well tolerated.

The lesions resolved completely within approximately one week. No secondary complications were observed. At follow-up, the patient remained completely asymptomatic, with no development of PHN.

## Discussion

The complete absence of pain was the most unusual feature of this case. Despite a characteristic unilateral thoracic dermatomal eruption, the patient reported no spontaneous pain, burning, dysesthesia, or tenderness, with pain consistently rated 0/10 throughout the acute episode and follow-up. This contrasts with the typical presentation of HZ, in which neuropathic pain is a prominent clinical feature. Painless HZ has nevertheless been described in the literature, including in elderly patients. A recent case report described an elderly man with a painless HZ eruption, emphasizing that the absence of pain does not exclude the diagnosis [6]. Our patient adds a different clinical context. She was 88 years old and had advanced CKD but no known immunosuppressive treatment, diabetes, or neurological disease.

The association between CKD and HZ is clinically relevant. CKD is characterized by complex alterations of both innate and adaptive immunity, including impaired immune responses and features of immunological dysfunction [4]. A case-control study of patients with CKD found that advanced CKD was common among patients with HZ, while dialysis and previous immunosuppressive treatment were associated with HZ occurrence [5]. Importantly, our patient was neither receiving immunosuppressive medication nor undergoing dialysis. Advanced CKD may therefore have represented a relevant background factor, although a causal relationship cannot be established from a single case.

Age-related decline in VZV-specific cell-mediated immunity, often referred to as immunosenescence, is thought to contribute to the increased incidence of HZ in older adults [1,2]. European literature also emphasizes the strong age dependence of HZ incidence and complications [2]. CKD may further alter immune responses through multiple mechanisms [4]. These factors may have contributed to the occurrence of HZ in our patient, but they do not explain with certainty why the eruption was completely painless.

The mechanism underlying painless HZ remains uncertain. One possible explanation is altered sensory processing associated with advanced age. Another possibility is subclinical peripheral sensory dysfunction related to severe renal disease. Uremic neuropathy is a recognized complication of advanced renal failure and predominantly affects peripheral nerves [7]. However, our patient had no known neurological disorder and did not undergo formal neurological testing for subclinical neuropathy. Therefore, attributing her painless presentation specifically to uremic neuropathy would be speculative.

The diagnosis of HZ is usually clinical when the eruption has a typical unilateral dermatomal distribution and characteristic vesicular morphology. Laboratory confirmation is particularly useful when the presentation is atypical or when alternative diagnoses are being considered. In this case, the morphology and distribution were considered sufficiently characteristic to support a clinical diagnosis. Nevertheless, the lack of VZV PCR confirmation is an important limitation. In future cases of unusually painless HZ, particularly in patients with relevant comorbidities or atypical clinical features, confirmatory testing for VZV should be considered when available to strengthen diagnostic certainty and facilitate documentation and further research.

Antiviral treatment is most effective when initiated early in the course of HZ. Valacyclovir is an established oral antiviral treatment for HZ and has been shown to provide effective treatment with convenient dosing. [8] Because valacyclovir is predominantly renally eliminated, dose adjustment is necessary in patients with severe renal impairment. The patient received a renally adjusted regimen of 500 mg once daily for seven days, which was well tolerated.

The absence of pain did not prevent recognition in this case because the cutaneous distribution was characteristic. This emphasizes an important practical lesson. Clinicians should not require pain to be present before considering HZ in an elderly patient with a unilateral dermatomal vesicular eruption.

This report has several limitations. First, VZV PCR confirmation was not performed because the clinical diagnosis was considered sufficiently characteristic during routine care. Although clinical diagnosis is generally appropriate in typical presentations, the unusual absence of pain would make virological confirmation particularly valuable in similar future cases. Second, the patient's vaccination status was unknown. Third, the dermatome was clinically estimated as approximately T9 rather than confirmed by formal neurological testing. Finally, because this is a single case, no causal relationship can be established between advanced CKD and the painless phenotype.

## Conclusions

HZ may present without pain, even in very elderly patients with advanced CKD who are not receiving immunosuppressive therapy. The absence of pain should therefore not exclude HZ when a characteristic unilateral dermatomal vesicular eruption is present. Careful clinical examination remains essential for recognizing atypical presentations and initiating appropriate treatment. When available, confirmatory VZV testing, preferably by PCR, should be considered in similarly unusual presentations to strengthen diagnostic certainty and improve clinical documentation.
